# A Multimodal Preclinical Assessment of MDMA in Female and Male Rats: Prohedonic, Cognition Disruptive, and Prosocial Effects

**DOI:** 10.1089/psymed.2023.0049

**Published:** 2024-06-17

**Authors:** Abshir S. Adam, Kayleigh S. LaMalfa, Yasaman Razavi, Stephen J. Kohut, Brian D. Kangas

**Affiliations:** Harvard Medical School, McLean Hospital, Belmont, Massachusetts, USA.

**Keywords:** MDMA, anhedonia, touchscreen cognition, social behavior, rats

## Abstract

**Background:**

Frontline antidepressants such as selective serotonin reuptake inhibitors (SSRIs) leave many patients with unmet treatment needs. Moreover, even when SSRIs reduce depressive symptoms, anhedonia, the loss of pleasure to previously rewarding activities, often remains unabated. This state of affairs is disheartening and calls for the development of medications to more directly treat anhedonia. The atypical psychedelic 3,4-methylenedioxymethamphetamine (MDMA) might have promise as a prohedonic medication given its efficacious applications for treatment-resistant post-traumatic stress disorder and comorbid depression. However, in addition to its prosocial effects as an entactogen, MDMA is also associated with neurotoxic cognitive deficits. The present studies were designed to examine the relative potency of MDMA in female and male rats across three distinct behavioral domains to assist in defining a preclinical profile of MDMA as a candidate prohedonic therapeutic.

**Methods:**

First, signal detection metrics of reward responsivity were examined using the touchscreen probabilistic reward task (PRT), a reverse-translated assay used to objectively quantify anhedonic phenotypes in humans. Second, to probe potential cognitive deficits, touchscreen-based assays of psychomotor vigilance and delayed matching-to-position were used to examine attentional processes and short-term spatial memory, respectively. Finally, MDMA's entactogenic effects were studied via pairwise assessments of social interaction facilitated by machine-learning analyses.

**Results:**

Findings show (1) dose-dependent increases in reward responsivity as quantified by the PRT, (2) dose-dependent deficits in attention and short-term memory, and (3) dose-dependent increases in aspects of prosocial interaction in male but not female subjects. Neither the desirable (prohedonic) nor undesirable (cognition disruptive) effects of MDMA persisted beyond 24 h.

**Conclusions:**

The present results characterize MDMA as a promising prohedonic treatment, notwithstanding some liability for short-lived cognitive impairment following acute administration.

## Introduction

Anhedonia, a blunted responsivity to previously rewarding stimuli, is a hallmark of several neuropsychiatric conditions, including major depressive disorder (MDD)^1^ and post-traumatic stress disorder (PTSD).^2^ It is noteworthy that for both MDD and PTSD, for which selective serotonin reuptake inhibitors are frontline treatments, anhedonia is typically unabated following standard regimens.^3,4^ Unfortunately, although the need for effective prohedonic medications is widely recognized, there currently are no approved pharmacotherapeutics to treat anhedonia. To that end, pursuits in preclinical and clinical drug development are actively focused on the discovery of new treatment strategies for anhedonia associated with MDD and PTSD.^5^

Given the current lack of prohedonic medications, recent efforts have revisited the potential efficacy of select psychoactive drugs more commonly associated with their recreational use than their potential value for treating psychiatric illness. One prominent success is the N-methyl-D-aspartate (NMDA) antagonist ketamine, which, despite its known abuse liability, was recently FDA-approved for use as a rapid-acting antidepressant in treatment-resistant populations.^6^ This approval was preceded by well-controlled studies, verifying its efficacy in laboratory animals^7–9^ and patient populations.^10^ Another timely example is the 5-HT2A agonist psilocybin, a psychedelic shown to significantly improve depression and anxiety in treatment-resistant patients,^11–13^ with efficacy that has recently been validated in Phase 2 clinical trials.^14^ A third example of considerable ongoing interest is 3,4-methylenedioxymethamphetamine (MDMA), a nonselective monoamine releaser and prototypical entactogen defined by its prosocial effects in humans and laboratory animals.^15,16^ Although perhaps best known as the club drug Ecstasy, promising findings from early uncontrolled experiments in the 1970s and 1980s^17^ have encouraged more rigorous empirical strategies in recent studies designed to examine its psychotherapeutic potential for neuropsychiatric disorders.^18,19^ Compelling results have already led the FDA to grant MDMA the status of a breakthrough therapy^20^ and, consequently, it currently is in Phase 3 clinical trials for use in treatment-resistant PTSD and comorbid depression.^21^ Like ketamine, these clinical successes are also supported by preclinical studies showing, for example, MDMA's ability to enhance fear memory extinction and modulate its reconsolidation^22–25^ and produce antidepressant-like effects^26^ in rodents. Despite its promise, however, the development of MDMA as a therapeutic has been slowed by evidence of undesirable effects such as neurocognitive toxicity^27–29^ and abuse liability.^30,31^ While such effects are unwanted, their impact on the clinical value of MDMA is unclear in the absence of a rigorous evaluation of its behavioral selectivity, that is, its relative potency in producing desirable and undesirable effects.

Given the clear need for medications to alleviate anhedonia in multiple psychiatric disorders and MDMA's uncertain behavioral profile, the purpose of this study was to characterize in rats MDMA's prohedonic effects and, at the same doses, evaluate its propensity for producing cognition-disruptive and prosocial effects. First, prohedonic effects were appraised using the Probabilistic Reward Task (PRT), an RDoC-recommended assay^32^ originally designed to objectively quantify reward responsivity in clinical populations^33^ and subsequently reverse-translated for rats,^34^ mice,^35^ and monkeys^36^ using touchscreen technology. In this task, subjects discriminate between one of two stimuli, and correct responses to one stimulus are probabilistically reinforced more often (rich alternative) than correct responses to the other (lean alternative). As predicted by signal detection theory,^37^ healthy subjects develop a response bias for the rich alternative. However, patients with anhedonia across a variety of clinical indications^38–41^ and rodents with a history of chronic stress or early-life adversity^42,43^ show a blunted response bias for the rich alternative, confirming the construct validity of this task. Second, MDMA was studied in touchscreen-based psychomotor vigilance and delayed matching-to-position tasks, which capture fundamental aspects of complex behavioral processes.^44^ These tasks were chosen to examine the effects of MDMA on attentional and short-term spatial memory processes, respectively, as deficits in these domains have previously been observed in human subjects following acute MDMA treatment.^45–48^ Third, in view of MDMA's well-known entactogenic effects,^15,16^ prosocial behavior was characterized using machine-learning analyses. This is a relatively novel approach to studying social behavior. As such, it was expected to provide useful data to supplement information regarding MDMA's desirable (prohedonic) and undesirable (cognition-disruptive) effects in the present studies.

## Methods

### Subjects

Forty adult Long-Evans rats (20 females, 20 males) obtained from Charles River Laboratories (Wilmington, MA) weighing between 150–175 (females) and 175–200 (males) grams were housed in a climate-controlled vivarium with a 12-h light/dark cycle. To establish sweetened condensed milk as a reinforcer for the touchscreen studies, female and male subjects were restricted daily to, respectively, ∼7–10 and 10–15 g of rodent chow given daily after the experimental session. Subjects in the observational study were not food restricted. All subjects had unrestricted access to water in their home cage. The protocol was approved by the Institutional Animal Care and Use Committee at McLean Hospital in accordance with established guidelines.^49^

### Touchscreen studies

#### Apparatus

Details and schematics of the experimental chamber have been previously published.^50^ Briefly, the right-hand wall of a 25 × 30 × 35 cm Plexiglass chamber was equipped with a 17″ touch-sensitive screen (1739L; ELO TouchSystems, Menlo Park, CA), above which a speaker bar (NQ576AT; Hewlett-Packard, Palo Alto, CA) was mounted. The chamber was housed in a light and sound-attenuating enclosure (40 × 60 × 45 cm). An infusion pump (PHM-100-5; Med Associates, St. Albans, VT) located outside the enclosure delivered a sweetened condensed milk solution (Sysco Corporation, Houston, TX) into the reservoir of a custom-designed aluminum receptacle (4 × 5 × 1 cm) mounted 2 cm above the floor bars on the center of the left-hand wall. Subjects were trained to respond on the touchscreen using previously published protocols.^34^ Scheduled rewards during the touchscreen studies consisted of 0.1 mL of 30% sweetened condensed milk, paired with an 880 ms yellow screen flash and 440 Hz tone. All experimental events and data collection were programmed in E-Prime Professional 2.0 (Sharpsburg, PA).

### Probabilistic Reward Task; *n* = 12, 6/sex

**Fig. 1.**
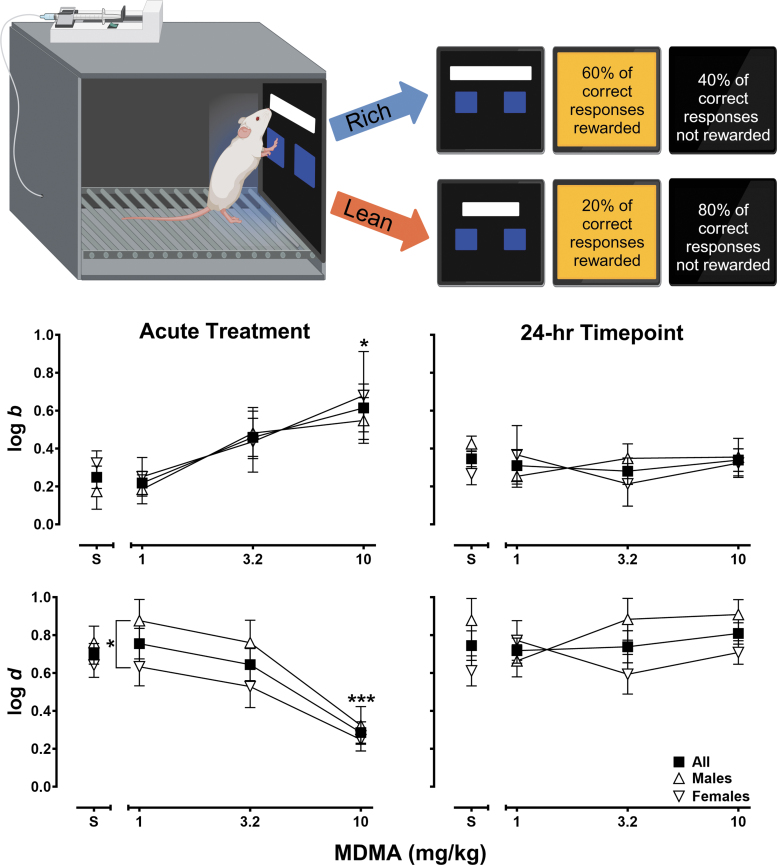
Probabilistic reward task schematic (top). Effects of saline and MDMA (1–10 mg/kg) on response bias (log *b*, upper graph panels) and task discriminability (log *d*, lower graph panels) following an acute 15-min pretreatment interval (left graph panels) and 24-h later (right graph panels). Squares represent mean (±SEM) data from all subjects (*n* = 12), triangles represent mean (±SEM) data from male subjects (*n* = 6), and inverted triangles represent mean (±SEM) data from female subjects (*n* = 6). *p < 0.05, ***p < 0.001. MDMA, 3,4-methylenedioxymethamphetamine.

Trials began with presentation of a white line on a black background, with its lower edge presented 3 cm above 5 × 5 cm left and right blue response boxes. The length of the line was either 600 × 120 px (31.5 × 6.5 cm: long line) or 200 × 60 px (10.5 × 3.25 cm: short line). Long and short line-length trial types varied in a quasi-random manner across 100-trial sessions such that there were exactly 50 trials of each type. Subjects were differentially reinforced to respond to the left or right response box depending on the length of the white line (long line: respond left, short line: respond right, or vice versa, counterbalanced across subjects). Each correct response was reinforced as described earlier and followed by a 5-s blackout period, whereas each incorrect response immediately resulted in a 10-s blackout period. Discrimination training sessions continued until accuracies for both line length trial types were ≥80% correct for two consecutive sessions, concordant with the performance criteria in previous human PRT studies.^33,38–41^ After this criterion was met, MDMA effects on PRT performance were examined by exposing subjects to a weekly four-session (Mon–Thurs) drug testing protocol as follows: training sessions on Mon and Tue were conducted in which all correct responses were rewarded. On Wed and Thurs, test sessions were conducted using 3:1 probabilistic reinforcement contingencies (see Fig. 1, top panel schematic), such that a correct response to one of the line lengths (long or short) was reinforced 60% of the time (rich stimulus), whereas a correct response to the other line length was reinforced 20% of the time (lean stimulus). Incorrect responses were never reinforced. Vehicle or MDMA (1, 3.2, 10 mg/kg) was administered in a mixed order 15 min before Wednesday's session and, consequently, 24 h before Thursday's test session.

### Titrating psychomotor vigilance task (*n* = 8, 4/sex)

**Fig. 2.**
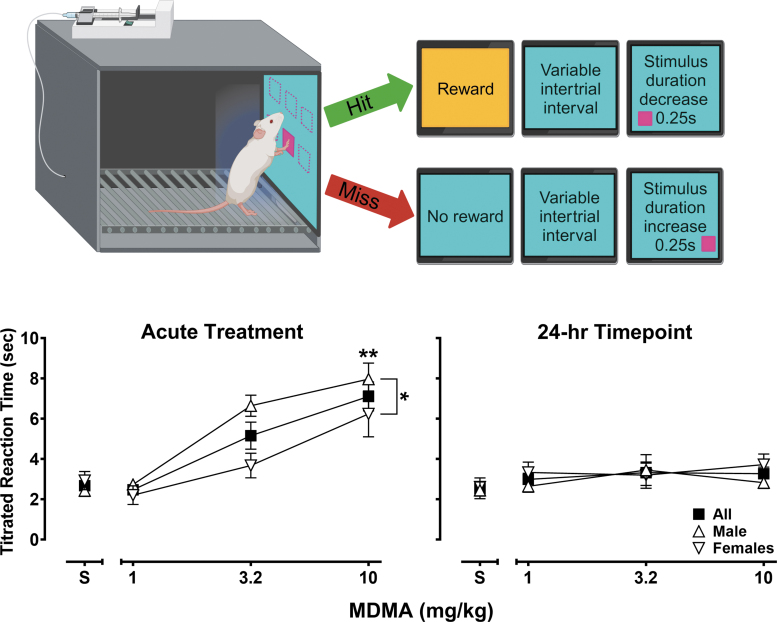
Titrating psychomotor vigilance task schematic (top). Effects of saline and MDMA (1–10 mg/kg) on titrated reaction times (s) following an acute 15-min pretreatment interval (left graph panel) and 24 h later (right graph panel). Squares represent mean (±SEM) data from all subjects (*n* = 8), triangles represent mean (±SEM) data from male subjects (*n* = 4), and inverted triangles represent mean (±SEM) data from female subjects (*n* = 4). *p < 0.05, **p < 0.01.

The titrating psychomotor vigilance task (tPVT) is a touchscreen assay, adapted here for rats, designed to examine sustained attentional processes.^51^ Daily sessions consisted of 50 trials and began with the presentation of a 7 × 7 cm pink square (target stimulus) on a blue background. The target stimulus was presented in one of six locations across the touchscreen, evenly spaced on a 3 × 2 matrix (see Fig. 2, top panel schematic). Both the location of the target stimulus and intermittence of its presentation (after 15, 30, or 45 s) were randomized across trials. If the subject successfully attended to the screen and touched the stimulus within the 10 s reaction timeframe, reinforcement was delivered as described earlier and the duration of the stimulus presentation on the next trial decreased by 0.25 s. If a subject failed to attend and respond to the target stimulus within the reaction timeframe, the stimulus disappeared without reward and its duration was increased by 0.25 s on the following trial. The stimulus duration during the first trial of the first session was set to 10 s and, thereafter, was set to the value of the last trial in the previous session. These titrating contingencies were designed to capture the attentional abilities of the subject across 50-trial sessions to examine the effects of MDMA on reaction time. Steady-state task performance was obtained before MDMA administration and was defined as five consecutive session-wide titrated reaction times means within ±20% of the average means of the five sessions. Thereafter, saline or doses of MDMA (1, 3.2, 10 mg/kg) were administered 15 min before a tPVT session in a mixed order no more than once per week.

### Titrating delay matching-to-position (*n* = 8, 4/sex)

**Fig. 3.**
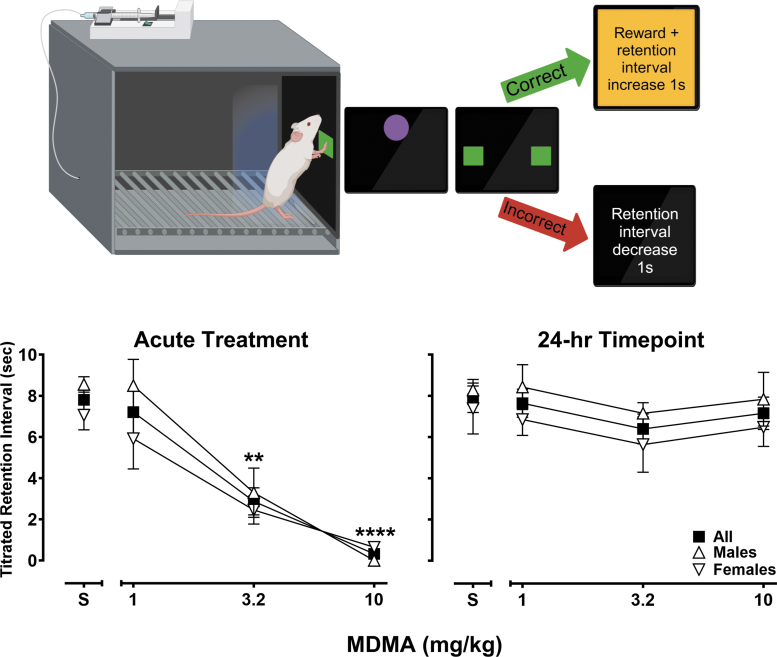
Titrating delayed matching-to-position task schematic (top). Effects of saline and MDMA (1–10 mg/kg) on titrated retention intervals (s) following an acute 15-min pretreatment interval (left graph panel) and 24 h later (right graph panel). Squares represent mean (±SEM) data from all subjects (*n* = 8), triangles represent mean (±SEM) data from male subjects (*n* = 4), and inverted triangles represent mean (±SEM) data from female subjects (*n* = 4). **p < 0.01, ****p < 0.0001.

The titrating delayed matching-to-position (tDMTP) task is a touchscreen assay, adapted here for rats, designed to examine short-term spatial memory.^52^ Daily sessions consisted of 48 trials and began with presentation of one 5 × 5 cm green box (sample stimulus) on a black background, positioned either far-left or far-right edge of the touchscreen 7.5 cm above the floor bars (see Fig. 3, top panel schematic). After making a response to the sample stimulus, the stimulus disappeared, and a retention interval was initiated during which a 5 × 5 cm purple circle was presented in the center of the touchscreen 15 cm above the floor bars. Responses to the retention interval stimulus (purple circle) were arranged during the retention interval to require the subject to recenter its position in the chamber before presentation of the comparison stimuli. The first response to the centered stimulus after the retention interval elapsed resulted in its disappearance and the presentation of two 5 × 5 cm green boxes on both the far-left and far-right of the touchscreen (comparison stimuli). A correct response to the comparison stimulus that matched the position of the previously presented sample stimulus resulted in reward delivery as described earlier and a 10 s blackout period. An incorrect response to the other comparison stimulus resulted in a 20 s blackout period. The retention interval was set to 0 s during the first trial of each session. For every two consecutive correct matches, the retention interval increased by 1 s on the following trial, whereas each mismatch decreased the retention interval by 1 s. These titrating contingencies were designed to capture the memorial abilities of the subject to examine the effects of MDMA on short-term spatial memory. Steady-state task performance was obtained before MDMA administration and was defined as five consecutive session-wide titrated retention interval means within ±20% of the average means of the five sessions. Thereafter, saline or doses of MDMA (1, 3.2, 10 mg/kg) were administered 15 min before a tDMTP session in a mixed order no more than once per week.

### Prosocial observation studies (*n* = 12, 6/sex)

#### Apparatus

**Fig. 4.**
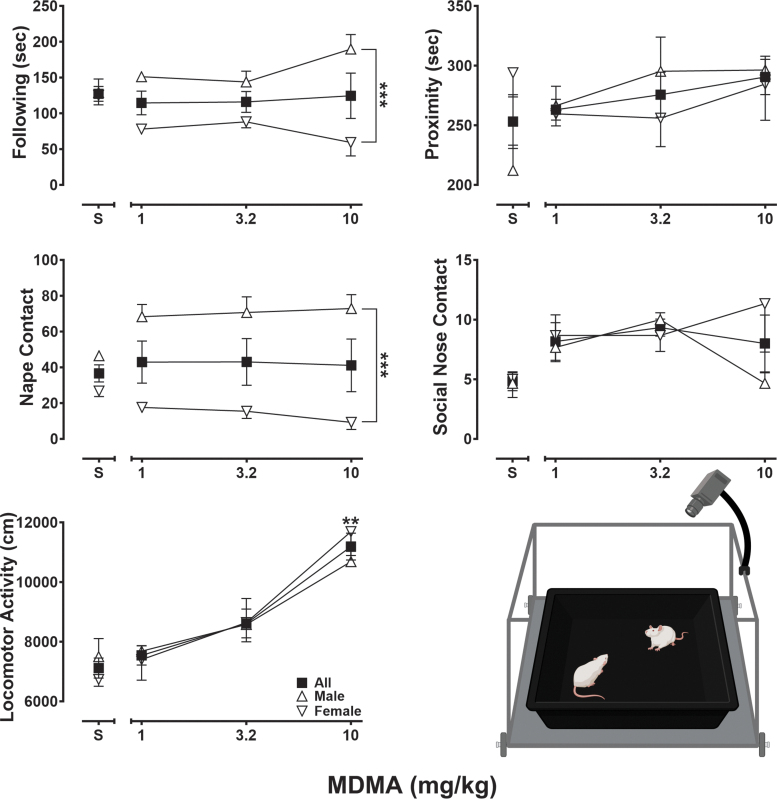
Effects of saline and MDMA (1–10 mg/kg) following an acute 15-min pretreatment interval and during a 10-min observational session on seconds of following (upper left graph panel), seconds of proximity (upper right graph panel), number of nape contacts (middle left graph panel), number of social nose contacts (middle right graph panel), and centimeters of locomotor activity (lower-left graph panel), observation arena schematic (lower-right panel). Squares represent mean (±SEM) data from all pairwise subjects (*n* = 12), triangles represent mean (±SEM) data from pairwise male subjects (*n* = 6), and inverted triangles represent mean (±SEM) data from pairwise female subjects (*n* = 6). **p < 0.01, ***p < 0.001.

A custom-designed observation arena was used to examine social interaction. The arena was constructed of half-inch black polycarbonate sheets and measured 60 × 60 × 30 cm. Sessions were videotaped using a GoPro 8 camera (San Mateo, CA) that was mounted 110 cm above the center of the arena to capture a complete overhead view (see Fig. 4, lower-right panel schematic).

#### Behavioral assessments

To evaluate the effects of MDMA on measures of prosocial behavior at the same doses evaluated for their prohedonic and cognition-disruptive effects, an unconditioned observational procedure was utilized that previously has been shown to be sensitive to MDMA's entactogenic effects in rodents.^53^ A 13-day protocol was used in which pairs of rats were initially administered saline on days 1, 3, and 5 to acclimate them to the experimental procedure and arena. The final saline session also served as baseline for comparison with MDMA. On days 7, 10, and 13, rats received one of three doses of MDMA (1, 3.2, 10 mg/kg) in a mixed order. Rat pairs were both injected with saline or the same dose of MDMA and placed individually into a clean home cage for the 15-min pretreatment interval. Each rat was then paired with an unfamiliar conspecific in the observation chamber for a 10-min social interaction observation. While in the arena, subjects could freely move and interact with one another, allowing for assessment of a diverse range of observable behavior.

### Drug

(±)-MDMA hydrochloride was provided by the National Institute on Drug Abuse Drug Supply Program (Rockville, MD). It was dissolved in 0.9% saline solution and administered via intraperitoneal injection. Drug doses (1–10 mg/kg) are expressed in terms of their free base weights.

### Data analysis

#### Probabilistic reward task

The implementation of probabilistic contingencies yields two primary dependent measures: response bias and discriminability, which can be quantified using equations derived from signal detection theory^37^ by examining the number of _Correct_ and _Incorrect_ responses in Rich and Lean trial types.

Response Bias is calculated using the following *log b* equation:

$$\log b=0.5\times log\left(\frac{\left(Rich_{Correct}+0.5\right)\times \left(Lean_{Incorrect}+0.5\right)}{\left(Rich_{Incorrect}+0.5\right)\times \left(Lean_{Correct}+0.5\right)}\right)$$

Task discriminability is calculated using the following *log d* equation:

$$\log d=0.5\times log\left(\frac{\left(Rich_{Correct}+0.5\right)\times \left(Lean_{Correct}+0.5\right)}{\left(Rich_{Incorrect}+0.5\right)\times \left(Lean_{Incorrect}+0.5\right)}\right)$$

#### Titrating psychomotor vigilance task

The primary dependent measure was mean titrated reaction time. This was computed by averaging the reaction time across each of the 50 trials that comprised a session.

#### Titrating delay matching-to-position

The primary dependent measure was mean titrated retention interval. This was computed by averaging the retention interval across each of the 48 trials that comprised a session.

#### Prosocial observational studies

Sessions were scored using DeepLabCut (DLC) v2.2.2, a multi-subject toolbox based on transfer learning with deep neural networks that has been shown to have a high prediction accuracy for assessing animal pose estimation compared with human observation.^54^ DLC obtained coordinates of defined body parts of both rats' snout, right ear, left ear, shoulder, cervical (spine 1), thoracic (spine 2), lumbar (spine 3), sacral (spine 4), and proximal (tail base), first (tail 1), second (tail 2), and distal caudal vertebra (tail end). Each body part was labeled on video frames to train the network^55^ using the following network training parameters: residual network “resnet 50” as the training algorithm, 120 labeled frames with a training fraction of 0.95, batch size of 8, and maximum iterations of 200,000. DLC's output file was post-processed in MATLAB to obtain quantitative measures of social interactions. Social behaviors of interest were operationally defined as *Following*: one rat's snout being within one tail length of the other rat's tail base with their body oriented in the same direction; *Proximity*: both rats being within one tail length of each other; *Nape Contact:* one rat's snout directed at the other rat's back region (spine 1); and *Social Nose Contact*: both rats' snouts within one body width of the other rat's tail base. These selected behaviors have previously been described as indicators of social attraction and interest.^56^ In addition, *Locomotor Activity*: total distance that rats traveled (cm), was used to quantify the psychomotor stimulant effects of MDMA.

#### Statistics

All data (PRT, tPVT, tDMTP, social interaction) were subject to two-way repeated measures analysis of variance with a Greenhouse-Geisser correction and dose and sex as factors. When appropriate, they were followed by Bonferroni's multiple-comparisons *post hoc* tests to examine the statistical significance of performance changes following doses of MDMA compared with saline treatment. The criterion for significance was set at *p* < 0.05. Statistical analyses were performed using Graph Pad Prism 9 (La Jolla, CA).

## Results

### Probabilistic reward task

All subjects learned to engage with the touchscreen and reached line-length discrimination training criterion following, on average, 12.5 (±1.1) sessions of training (males 10.3 [± 0.7], females: 14.7 [± 1.7]). Figure 1 presents the effects of saline and MDMA (1–10 mg/kg) on response bias (log *b*, upper panels) and task discriminability (log *d*, lower panels) following acute treatment (left panels) and 24-h post-administration (right panels). PRT outcomes are presented across all subjects (squares, *n* = 12) and by sex (males, *n* = 6, triangles; females, *n* = 6, inverted triangles). As shown in the upper-left panel, following administration of saline, prototypical log *b* values of ∼0.2–0.3 were observed. Administration of MDMA produced significant dose-dependent increases in log *b* (*F*[1.98, 19.77] = 3.89, *p* = 0.04) that did not differ by sex (*F*[1, 10] = 0.67, *p* = 0.43). As the lower-left panel shows, significant decreases in log *d* were also observed (*F*[1.89, 18.90] = 9.14, *p* = 0.002), as were significant sex differences with male subjects showing, on average, higher levels of task discriminability following treatment with saline and MDMA (*F*[1, 10] = 7.40, *p* = 0.02). As the right panels of Figure 1 show, both response bias (upper right panel) and task discriminability (lower right panel) returned to levels that closely approximated vehicle during sessions conducted 24 h post-MDMA administration (log *b*, *F*[2.11, 21.12] = 0.21, *p* = 0.83; log *d*, (*F*[2.22, 22.24] = 0.59, *p* = 0.58). The absence of significant sex differences was also confirmed statistically in log *b* (*F*[1, 10] = 0.70, *p* = 0.42) and log *d* (*F*[1, 10] = 2.65, *p* = 0.14) during the 24-h timepoint; however, a dose by sex interaction in log *d* emerged (*F*[3, 30] = 3.26, *p* = 0.04).

### Titrating psychomotor vigilance task

All subjects learned to engage with the touchscreen and reached the tPVT performance criterion on average following 19.6 (±2.4) sessions of training (males 19.0 [±1.5], females: 20.3 [±4.9]) with titrated reaction time values of 3.0 (±0.3) seconds (males 2.9 [± 0.3], females: 3.1 [±0.5]). Figure 2 presents the effects of saline and MDMA (1–10 mg/kg) on session-mean titrated reaction time values, with outcomes presented across all subjects (squares, *n* = 8) and by sex (males, *n* = 4, triangles; females, *n* = 4, inverted triangles). Performance following administration of saline closely approximated that of baseline values; however, as the left panel shows, significant dose-related increases in titrated reaction time values were observed following MDMA treatment (*F*[1.82, 10.93] = 23.92, *p* = 0.0001). In addition, although performance during baseline and following saline administration was highly similar between sexes, MDMA's dose-related deficits in male subjects were significantly more pronounced relative to those in females (*F*[1, 6] = 8.13, *p* = 0.03). As the right panel shows, however, performance during sessions conducted 24-h post-MDMA administration returned to vehicle levels following all doses (*F*[1.65, 9.91] = 2.09, *p* = 0.18) and in both sexes (*F*[1, 6] = 0.42, *p* = 0.54).

### Titrating delay matching-to-position

All subjects learned to engage with the touchscreen and reached the tDMTP performance criterion following, on average, 40.6 (±3.2) sessions of training (males 42.8 [±6.6], females: 38.5 [±1.3]) with titrated retention intervals of 7.3 (±0.5) seconds (males 7.5 [±0.6], females: 7.2 [±0.8]). Figure 3 presents the effects of saline and MDMA (1–10 mg/kg) on session-mean titrated retention intervals, with outcomes presented across all subjects (squares, *n* = 8) and by sex (males, *n* = 4, triangles; females, *n* = 4, inverted triangles). As the left panel shows, following treatment with saline, average retention intervals ∼8 s; performance in males (∼8.5 s) was somewhat better than that of females (∼7 s). Administration of MDMA produced dose-dependent deficits in the tDMTP task (*F*[1.08, 6.46] = 27.35, *p* = 0.0014), with sex differences in MDMA-induced deficits that trended toward statistical significance (*F*[1, 6] = 5.75, *p* = 0.053). Performance deficits in this task also were not evident during sessions conducted 24 h after MDMA administration (*F*[2.25, 13.51] = 1.34, *p* = 0.30) nor did they differ by sex (*F*[1, 6] = 1.58, *p* = 0.26) at this time point.

### Prosocial observational studies

Figure 4 presents various observable aspects of rodent social behavior and locomotor activity after administration of saline or MDMA (1–10 mg/kg), with outcomes presented across all pairwise subjects (squares, *n* = 12) and by sex (male pairs, *n* = 6, triangles; female pairs, *n* = 6, inverted triangles). As the upper-left panel shows, male and female rats spent on average 127 (±10) seconds following each other after saline administration. Although following behavior did not differ statistically by dose (*F*[1.55, 6.21] = 0.33, *p* = 0.68), it did differ by sex (*F*[1, 4] = 75.32, *p* = 0.001) and showed a dose by sex interaction (*F*[3, 12] = 5.80, *p* = 0.01). In male subjects, MDMA treatment was associated with dose-dependent increases in following behavior; however, in stark contrast, female subjects displayed dose-related decreases in following behavior. As the upper-right panel shows, average time spent in proximity following saline administration was 212 (±21) for males and 294 (±20) seconds for females. In male rats, the total time spent in proximity increased in a dose-dependent manner, whereas in females, MDMA treatment produced modest decreases in time spent in proximity relative to saline across the range of doses studied. However, proximity did not differ statistically by dose (*F*[1.42, 5.68] = 1.06, *p* = 0.38) or sex (*F*[1, 4] = 0.20, *p* = 0.68). As shown in the middle-left panel, MDMA treatment produced significantly different effects in the number of nape contacts by sex (*F*[1, 4] = 229.10, *p* = 0.0001) and showed a dose by sex interaction (*F*[3, 12] = 5.75, *p* = 0.01), but did not differ statistically by dose (*F*[2.07, 8.27] = 0.57, *p* = 0.59). Although more nape contacts following saline administration were observed in male subjects (46 [ ± 3]) relative to female subjects (27 [±2]), MDMA increased those levels in males and decreased them in females, across all doses tested. As the middle-right panel shows, social nose contact was low following saline administration (∼5 [±0.5] counts in males and females) and, although elevated values were observed in both sexes following MDMA treatment, these findings did not differ by dose (*F*[2.11, 8.43] = 2.21, *p* = 0.17) or sex (*F*[1, 4] = 0.77, *p* = 0.43). Finally, as presented in the lower-left panel, the total distance traveled during observation sessions in which rats received saline was 7498 (±605) and 6735 (±225) cm for male and female rats, respectively. Consistent with MDMA's psychomotor stimulant properties, the total distance traveled increased significantly in a dose-dependent manner (*F*[2.32, 9.29] = 17.22, *p* = 0.0006) but did not differ by sex (*F*[1, 4] = 0.0002, *p* = 0.99).

## Discussion

The present studies provide a preclinical profile of MDMA's action across diverse aspects of rodent behavior. Signal detection outcomes from the PRT confirm that MDMA enhances reward responsivity, evident in dose-related increases in response bias for the more richly rewarded stimulus. The magnitude of effect following 10 mg/kg MDMA in this reverse-translated task approximates that previously observed with the rapid-acting antidepressants scopolamine in rats^34^ and ketamine in marmoset monkeys.^36^ MDMA's dose-related increases in log *b* also resembled previous rat PRT findings following *d*-amphetamine treatment.^34^ As such, given their overlapping pharmacology, it is possible that, like *d*-amphetamine, MDMA's prohedonic efficacy might be mediated via increased striatal dopamine transmission. Indeed, the prohedonic actions of *d*-amphetamine are proposed to play a role in its reported antidepressant effects,^57^ although well-controlled clinical investigations are needed. Moreover, this would be consistent with converging evidence of dopamine as a pivotal modulator of reward learning in humans^58–61^; however, this has yet to be directly confirmed under these conditions. MDMA's peak effect on reward responsivity was accompanied by significant decrements in task discriminability. Although log *b* and log *d* were modified by MDMA in a comparable dose-dependent manner, these signal detection metrics are independent from each other. Indeed, MDMA's profile is similar to that observed with scopolamine in this regard, but not *d*-amphetamine and ketamine, which showed dose-dependent increases in log *b* without significant changes in log *d*. Despite mounting preclinical and clinical evidence of MDMA's promise in psychotherapeutic approaches to PTSD and MDD, its prohedonic efficacy had not been previously assessed. This is similar to the development of ketamine in that confirmation of its anti-anhedonic effects was documented^62,63^ long after its antidepressant effects were confirmed.^64^ Because the rodent PRT is reverse-translated from the human laboratory, systematic replication of these prohedonic MDMA effects can be readily tested in patient populations in the manner already conducted for other drugs.^65^

In the context of medications development, it is interesting and important to systematically examine both the desirable (prohedonic) and unwanted (cognition disruptive) effects of candidate pharmacotherapeutics. In this regard, MDMA also had dose-related cognition-disruptive effects in animal models designed to examine attentional processes and short-term spatial memory, with statistically significant disruption observed following both 3.2 and 10 mg/kg. Importantly, all effects of MDMA on cognitive performance—both in signal detection metrics and in measures of attention and short-term memory—were absent during sessions conducted 24 h after MDMA administration. The present findings of MDMA's cognition-disruptive effects in rodents are consistent with previous human studies showing performance deficits in attention and memory tasks following acute MDMA treatment.^45–48^ The rapid offset of these deleterious effects is also in accord with its half-life of <8 h in humans^66^ and rats.^67^ The extent to which MDMA's lack of behavioral selectivity may constrain its therapeutic application is currently unclear and depends, to a great extent, on the magnitude of such cognitive disturbances in human subjects. It is again instructive to consider the development of ketamine, which has profound dissociative effects following acute administration in therapeutic settings. Indeed, an early trial documented memory impairment following a single ketamine infusion in treatment-resistant patients,^68^ which bred concern that ketamine's therapeutic efficacy might be accompanied by similar cognitive impairment as documented with its recreational use.^69^ Subsequent studies, however, observed either no impairment^70,71^ or evidence of procognitive effects,^72–75^ putatively via the reduction of depressive symptoms following treatment regimens. It is possible that MDMA's side effects similarly will not encumber its therapeutic application.

Regarding MDMA's entactogenic effects, significant increases in diverse aspects of social behavior were absent when data from both sexes were grouped together. However, some qualitative rather than quantitative sex differences were noted in pairwise interactions. While dose-related increases in certain aspects of prosocial behavior were evident in male subjects, the converse was observed in females. The reasons for these conspicuous sex differences in social interaction, as well as those observed in the PRT's log *d* and tPVT's titrated reaction time metrics are currently unclear. Sex differences in the physiological, neurochemical, and abuse-related effects of MDMA have also been previously reported in rats.^76–78^ In human studies, females were found to have higher subjective ratings of negative mood/anxiety, heightened thought disturbances, and increased fear of body control following MDMA administration compared with males,^79,80^ which may interfere with the expression of the entactogenic effects. It is interesting to note that in the present studies, MDMA produced significant dose-related increases in locomotor activity that were highly similar in both sexes. These psychomotor stimulant effects of MDMA were not surprising but confirm comparable *nonsocial* effects in males and females under the pairwise arena conditions arranged. Further, that females did not show increases in any aspect of prosocial behavior despite increased locomotor activity suggests that these behavioral effects of MDMA are separable. It is important to consider an alternative conclusion, however, namely that MDMA-induced prosocial behavior under the current conditions in rats is minimal at best. This would represent a prominent species difference but, nevertheless, would be consistent with previous studies in rats investigating the effects of MDMA on social behavior under a variety of other conditions.^81–83^ In the context of preclinical medications development, the present findings offer suggestive evidence that MDMA's therapeutic potential as a prohedonic is not mediated by its entactogenic effects, the mediator often assumed driving its clinical efficacy. If this dissociation proves to be a reliable outcome, documenting prosocial phenotypes in rats may not necessarily serve as therapeutically predictive outcomes.

The present studies are not without limitations. First, given the acute dosing protocol in these studies, determining the effects of chronic MDMA exposure, and whether tolerance would develop to either the desirable or undesirable effects documented here, would enhance characterization of MDMA's preclinical profile and viability as a prohedonic medicine. Second, given MDMA's nonselective monoaminergic mechanism of action, the relative contribution of dopamine and serotonin release to the various behavioral outcomes is unknown. Future work examining the ability of selective antagonist pretreatments to block prohedonic or cognition-disruptive effects would provide important mechanistic clues. Finally, it is important to note that because these studies were conducted in healthy rats, insomuch as no chronic stressors were programmed, any conclusions must be limited to MDMA's prohedonic, rather than anti-anhedonic, potential. Future studies examining MDMA's ability to rescue anhedonic phenotypes in the PRT (i.e., blunted log *b* values), as already documented to occur following rodent models of social defeat^42^ and early-life adversity,^43^ would, in turn, appraise its anti-anhedonic efficacy and time course and perhaps serve as a closer preclinical surrogate to the clinical condition.

Taken together, the present findings underscore goals for the development of prohedonic drugs, namely, by retaining MDMA-like prohedonic efficacy and reducing the likelihood of cognition-disruptive effects. With regard to MDMA itself, it would be useful to compare the effects of MDMA's enantiomers inasmuch as they and their active metabolites differ in their monoaminergic activity.^84,85^ Such findings might provide insight into whether the beneficial and unwanted effects of MDMA can be dissected on a neuropharmacological basis. More generally, the present results with MDMA provide a standard against which to measure improvement in the preclinical profile of candidate medications. This may be an effective strategy to accelerate medications development for anhedonia in the variety of neuropsychiatric conditions in which it is prominent.
